# *Anaplasma* Infection in Ticks in Southeastern Region of Iran

**DOI:** 10.18502/jad.v14i2.3730

**Published:** 2020-06-30

**Authors:** Reza Ranjbar, Mehdi Anjomruz, Ahmad Ali Enayati, Mehdi Khoobdel, Atiyeh Rafinejad, Javad Rafinejad

**Affiliations:** 1 Molecular Biology Research Center, Systems Biology and Poisonings Institute, Baqiyatallah University of Medical Sciences, Tehran, Iran; 2 Department of Medical Entomology and Vector Control, School of Public Health, Tehran University of Medical Sciences, Tehran, Iran; 3 Department of Medical Entomology, School of Public Health and Health Sciences Research Centre, Mazandaran University of Medical Sciences, Sari, Iran; 4 Health Research Center, Baqiyatallah University of Medical Sciences, Tehran, Iran

**Keywords:** *Anaplasma*, Molecular, Ticks, Iran

## Abstract

**Background:**

Anaplasmosis and Ehrlichiosis are the most important tick-borne diseases. This study was conducted in three cities of Kerman Province in Iran to investigate the circulation of the bacteria in ticks collected from sheep.

**Methods:**

Ticks were collected from animals using Srkj forceps and transferred to the Entomology lab in cold chain. After specimen’s identification, they kept at −70 °C. Tick DNA was extracted using Bioneers DNA extraction kits followed by Nested PCR technique to amplify ribosomal 16S rRNA gene to detect *Anaplasma* infection in ticks.

**Results:**

472 sheep were examined from which 349 ticks were collected and identified in laboratory using valid keys. Tick specimens belonged to two genera and four species; *Hyalomma marginatum* (62.47%) was the most frequent and *Hylomma asiaticum* (5.73%) showed the least abundance. The infestation rate to different tick species was different in three regions of Kerman Province. Observation revealed that 24 specimens (58.3%) were positive for *Anaplasma*. There is a significant difference between male and female infection rate. However, there is no significant difference between these variables in each of these cities.

**Conclusion:**

This study shows high infection rates to Anaplasma in hard ticks. It is essential for health and veterinary authorities and farmers to use appropriate strategies to control ticks to reduce the infestation.

## Introduction

Ticks are mandatory blood-sucking ecto-parasites of vertebrate’s especially wild animals and only about 10 percent of its species feed from humans and livestock, particularly sheep and cow. Anaplasmosis usually occurs in tropical and subtropical regions and is reported from Africa, the Middle East, Asia, Australia, USA, South and Central America and southern Europe (1, 2, 3).

Ticks directly affect their host by causing irritation, allergic reactions, anemia, weight loss, paralysis and even death. Spotted fever, Rocky Mountains fever, Siberia tick typhus, tularemia, Lyme disease, relapsing fever, Crimean-Congo hemorrhagic fever, Anaplasmosis and Ehrlichiosis are among the most important tick-borne diseases transmitted to humans by ticks (4, 5, 6, 7). Ticks are also involved in transmitting certain diseases to livestock the most important of which include swine fever in Asia, viral hemorrhagic fever, anaplasmosis, theileriosis and babesiosis (8, 9, 10). Certain pathogens transmitted by ticks can be spread by contaminated airborne particles, hence, they can be used for military purposes (Bioterrorism), many diseases having bioterrorism potential are among diseases transmitted by arthropods including plague, yellow fever, tularemia, Rift Valley fever, Q fever, viral encephalitis, etc. The first practical use of insects was by the Japanese during World War II using ticks infected with plague agent (*Yersinia pestis*), *Musca domestica* with *cholerae* bacteria (vibrio *cholerae*). During World War II, the Russians used lice infected with typhus agent (*Rickttesia typhi*) against the Germans (11, 12, 13, 14, 15). Development of Genetics and production of arthropods with the ability to transmit life-threatening diseases like Human immunodeficiency virus infection and acquired immune deficiency syndrome (HIV/AIDS), high potency sting, resistance to pesticides and also production of biological agents with more virulence, drug resistance and stability at ambient conditions have greatly contributed to the development of bioterrorism (13) a fact that increases their importance (16). Anaplasmosis, a rickettsia zoonosis, is one such disease, among 30 new ones that have emerged since 1973. The rickettsia is a mandatory intracellular parasite of red blood cells. Infected red blood cells lose their functionality in circulatory system and are destroyed. This causes diseases in livestock and wild ruminants like cow, sheep, goats, and deer although this disease causes clinical symptoms only in cow and giraffes. The disease is transmitted through red blood cells of infected animals. When an animal becomes ill, despite its recovery, it remains as a source of infection forever. Anaplasmosis is transmitted to others hosts by ticks that have bitten infected animals. The pathogen will also multiply in infected ticks. Then infected ticks transmit the disease to healthy animals. The pathogen can be transmitted to healthy animals by mosquito bites or contaminated equipment with infected animal blood such as infected syringe or infected equipment of cutting horn as well (17, 18, 19, 20, 21). Anaplasmosis begins with a mild fever, but it gradually becomes more serious. Treatment is usually effective if it is provided early. Tetracycline and Amido propionate are two first line drugs used for treatment (18, 19, 22, 23). In Iran, the first case of this disease was reported in 2003 from north of the country. *Anaplasma* bacteria are detected in animal blood samples in provinces of Khorasan, Esfahan, Fars, Mazandaran and Sistan and Baluchistan (24, 25, 26, 27, 28). Considering the relatively high tick burden of animals and importance of Anaplasmosis and other tick-borne diseases in livestock as well as humans, determining the species of infecting bacteria and tick vectors and their infection rate are very important. This could be an effective step in diagnosis of diseases transmitted by ticks and development of strategies to preventing diseases by controlling ticks and patient treatment.

## Materials and Methods

### Study area

**Fig. 1.**
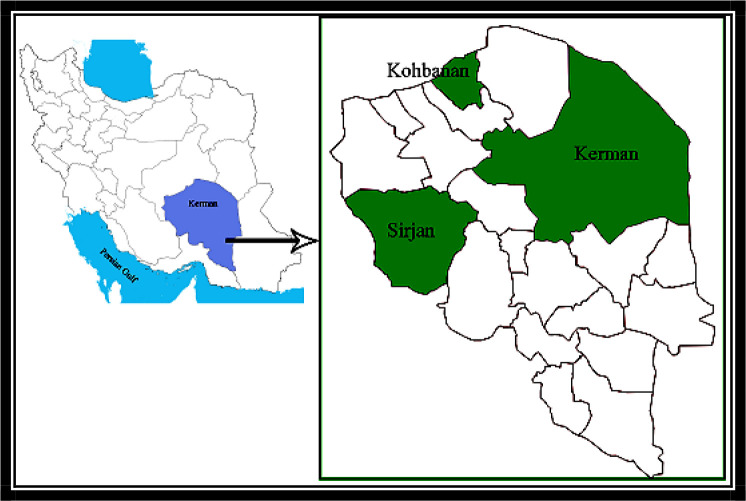
Geographical location of Kerman Province, Iran

This study is undertaken in three cities of Kerman Province including Kerman, Kohbanan and Sirjan. Kerman is located in the south-east of Iran central plateau, between 53° 26′ to 59° 29′ east longitude and 25° 55′ to 32° north latitude. Encompassing an area of more than 183/285 square kilometers and approximately eleven percent of the country’s area, Kerman is the largest province in Iran located in southeast of the country with a history of human settlement since four millennium BC. Kohbanan is located at 31°.42′ 36″ North and 56°.28′ 75″ East in the far northern parts of Kerman Province, southeast of Iran with a population in 2011 of 21721 people. Sirjan is another city of Kerman Province (29°.45′ 55°.67′) is located in the West of this province and has a population in 2011 of about 269000 (Fig. 1).

### Sample Collection

Ticks were separated from body of sheep, goat and cow (ear, groin, tail, back and neck) using Srkj forceps so as not to harm the ticks and animals. They were then put in appropriately labeled tubes and transferred to the Entomology lab in cold chain. After specimens identification, selected ticks were kept at −70 °C in micro tubes for PCR (29, 30).

### DNA extraction

Bioneers DNA extraction Kits (iNtRON Biotechnology, Korea) was used to extract DNA according to the instructions of the manufacturer. Nested PCR technique with primers designed by Rar et al. was used to amplify ribosomal 16S rRNA gene in order to detect *Anaplasma* infection in ticks (31). The primers are designed based on conserved and highly changeable region V1 of ribosomal gene 16S (16S rRNA) of *Anaplasma sp*. and *Ehrlichia sp*. producing DNA fragments of about 500bp (Table 1). Polymerase Chain Reaction (PCR) protocol consisted of 35 cycles of a preheating phase at 94 °C for 5min, denaturation at 94 °C for 1min, annealing at 57 °C for 1min, extension at 72 °C for 1min and a final incubation at 72 °C for 7min. Before finishing the first phase of PCR reaction, master mix of the second phase is prepared. In the second phase of the nested PCR, different primers are used (Table 1) and also two micro liter of (PCR) product is used as template and annealing was set at 54 °C. PCR products were kept in a refrigerator before electrophoresis using 1% agarose gel.

**Table 1.** List and details of primers used in this study

**Primer**	**Nucleotide sequences**
**Forward: Ehr1**	5′-GAACGAACGCTGGCGGCAAGC-3′
**Reverse: Ehr2**	5′-AGTATCCGAGACCAGATAGCCGC-3′
**Reverse: Ehr3**	5′-TGCATAGGAATCTACCTAGTAG-3′
**Reverse: Ehr4**	5′-CTAGGAATTCCGCTATCCTCT-3′

## Results

**Fig. 2.**
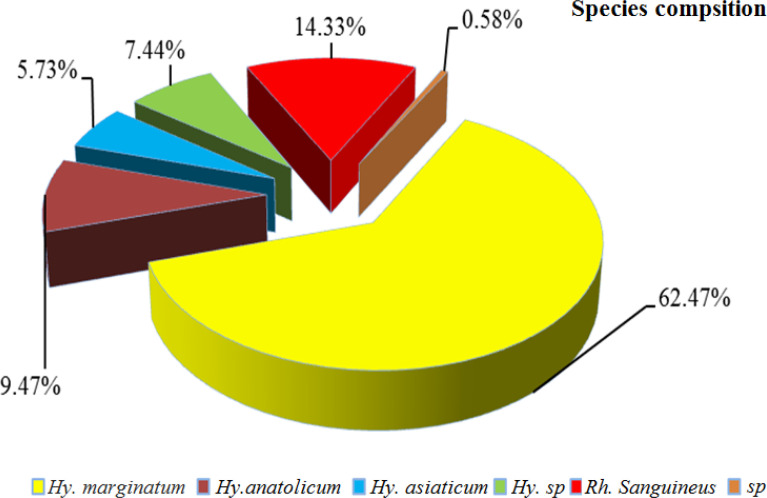
The infestation rate to different tick species in Kerman Province, southern Iran in 2015

During this study, a total of 472 sheep were examined for tick infestation in three cities of Kerman Province in spring 2015. A total of 349 ticks were collected from 154 animals resulting in a tick burden of 2.26. Collected ticks were identified in laboratory using valid diagnosis keys (32, 33). As presented in Fig. 2, collected Ixodidae tick specimens belonged to two genera and four species including *Hy. marginatum* (62.47%), *Hy. anatolicum* (9.47%), *Hy. asiaticum* (5.73%), *Hyalomma.sp* (7.44%), *Rhipicephalus sanguineus* (14.33%) and unidentified specimens (0.58 %) (Fig. 2).

Among identified species, *Hy. marginatum* with 62.47% showed the highest frequency and *Hy. asiaticum* (5.73%) the lowest (Fig. 2). Totally, from 349 tick specimens, 63 were collected in Kerman, 98 in Sirjan and 188 in Kohbanan. According to the results in Kerman, *Rh. sanguineus* with a prevalence of 10.05% showed the highest frequency and *Hy. anatolicum*, *Hy. marginatum* and *Hy. asiaticum* species with 3.43%, 2.58% and 0.29 % were in the next orders respectively. *Hyalomma marginatum*, *Hy. anatolicum*, *Rh. sanguineus* and *Hy. asiaticum* with 20.92, 2.87, 1.72 and 0.85 percent showed the highest frequency in Sirjan City respectively. *Hyalomma marginatum* with 39.97 percent had the highest frequency and *Hy. asiaticum*, *Hy. anatolicum* and *Rh. sanguineus* with 4.59, 3.15 and 2.56 percent showed the highest frequency in Kohbanan respectively (Table 2).

**Table 2.** Species composition in Kerman Province (A=Kohbanan and B=Sirjan and C=Kerman)

**Genus/Species**	**A B C province**			**N**_**T**_ **(100%)**

	**A**	**B**	**C**	
***Hy. marginatum***	136(38.97%)	73(20.92%)	9(2.58%)	218(62.47%)
***Hy. anatolicum***	11(3.15%)	10(2.87%)	12(3.43%)	33(9.45%)
***Hy*. *asiaticum***	16(4.59%)	3(0.85%)	1(0.29%)	20(5.73%)
***Hy. sp***	16(4.59%)	4(1.14%)	6(1.71%)	26(7.44%)
***Rh. sanguineus***	9(2.56%)	6(1.72%)	35(10.05%)	50(14.33%)
**unidentified sp.**	0(0%)	2(0.58%)	0(0%)	2(0.58%)
**Total**	188(53.86%)	98(28.08%)	63(18.06%)	349(100%)

**Table 3.** Infection rates of Anaplasmosis in ticks collected from Kerman Province, southern Iran in 2015

**City**	**Genus/Species**	**Nymph**	**Female (♀)**		**Male (♂)**		**N**_**P&N**_ **(100%)**		**N**_**T**_ **(100%)**

		**Negative**	**Positive**	**Negative**	**Positive**	**Negative**	**Positive**	**Negative**	
**City A**	*Hy. marginatum*	0(0%)	0(0%)	6(16.22%)	2(5.4%)	13(35.14%)	2(5.41%)	19(51.35%)	21(56.76%)
	*Hy. mnatoilicum*	0(0%)	0(0%)	2(5.4%)	0(0%)	4(10.82%)	0(0%)	6(16.22%)	6(16.22%)
	*Hy*. *msiaticum*	0(0%)	0(0%)	2(5.4%)	0(0%)	4(10.82%)	0(0%)	6(16.22%)	6(16.22%)
	*Hy. Sp*	0(0%)	0(0%)	1(2.7%)	0(0%)	0(0%)	0(%)	1(2.7%)	1(2.7%)
	*Rh. manguineus*	0(0%)	0(0%)	1(2.7%)	0(0%)	2(5.4%)	0(0%)	3(8.1%)	3(8.1%)
	**Total A**	**0(0%)**	**0(0%)**	**12(32.43%)**	**2(5.4%)**	**23(62.17%)**	**2(5.41%)**	**35(94.59%)**	**37(100%)**
**City B**	*Hy. marginatum*	0(0%)	2(5.71%)	5(14.29%)	5(14.29%)	10(28.57%)	7(20%)	15(42.86%)	22(62.86%)
	*Hy. anatoilicum*	0(0%)	0(0%)	2(5.71%)	0(0%)	4(11.43%)	0(0%)	6(17.14%)	6(17.14%)
	*Hy*. *asiaticum*	0(0%)	0(0%)	0(0%)	0(0%)	2(5.71%)	0(0%)	2(5.71%)	2(5.71%)
	*Hy. Sp*	0(0%)	0(0%)	2(5.71%)	0(0%)	0(0%)	0(0%)	2(5.71%)	2(5.71%)
	*Rh. Ssanguineus*	0(0%)	0(0%)	1(2.86%)	0(0%)	2(5.71%)	0(0%)	3(8.58%)	3(8.58%)
	**Total B**	**0(0%)**	**2(5.71%)**	**10(28.57%)**	**5(14.29%)**	**18(51.42%)**	**7(20%)**	**28(80%)**	**35(100%)**
**City C**	*Hy. marginatum*	0(0%)	1(4.17%)	0(0%)	1(4.17%)	3(12.5%)	2(8.33%)	3(12.5%)	5(20.83%)
	*Hy. mnatoilicum*	0(0%)	0(0%)	0(0%)	2(8.33%)	3(12.5%)	2(8.33%)	3(12.5%)	5(20.83%)
	*Hy*. *msiaticum*	0(0%)	0(0%)	0(0%)	1(4.17%)	0(0%)	1(4.17%)	0(0%)	1(4.17%)
	*Hy. Sp*	1(4.17%)	0(0%)	0(0%)	0(0%)	0(0%)	0(0%)	1(4.17%)	1(4.17%)
	*Rh. manguineus*	0(0%)	8(33.33%)	2(8.33%)	1(4.17%)	1(4.17)	9(37.5%)	3(12.5%)	12(50%)
	**Total C**	**1(4.17%)**	**9(37.5%)**	**2(8.33%)**	**5(20.84%)**	**7(29.16%)**	**14(58.33%)**	**10(41.67%)**	**24(100%)**
**Total ABC**		**1(1.05%)**	**11(11.45%)**	**24(25%)**	**12(12.5%)**	**48(50%)**	**23(23.95%)**	**73(76.05%)**	**96(100%)**

## Discussion

The results revealed that 23 out of 96 analyzed specimens were positives for *Anaplasma*; 11.45% *Hy. marginatum*, 2.08% *Hy. anatolicum*, 1.04% *Hy. asiaticum*, 0% *Hy. sp*, and 9.37% *Rh. sanguineus* were positives. Nested-PCR is a sensitive molecular technique to determine infection to *Anaplasma sp* in ticks collected from animals. In 1910, *Anaplasma marginale* was described first by Theiler in African cows’ Red Blood Cell (RBC) as a marginal spot (34); afterward, in 1911, he introduced *An. centrale*, a subspecies of *An. marginale* which is located in the center of Erythrocytes and has less pathogenicity (35). Lesto Kard, in 1924, demon-trated *An. ovis* in sheeps’ RBC. Donatien and Lestoquard observed *An. Bovis* in French cows’ RBC, during an experimental transmission of Theileria through *Hyalomma* Ticks (36). Dumler et al. (2001), after the modification of *Ehrlichia equi*, *Ehrlichia phgocytophila* species plus unknown factor of human *Ehrlichia granulocytic*, categoryzed them under a species named *An. Phgocytophilum* (37). From the amount of 60 cows, 391 sheep, and 385 goats, through a blood smear test observed that 19.37% of cows were infected to *An. marginale*, and 80.3% of sheep and 38.92% of goats were infected to *An. ovis*, during a study between 1999 and 2002 in Khorasan province in Iran by Razmi et al. (2005), there was no a significant difference between age and gender groups regarding the infection rate of *Anaplasma* and the amount of *Anaplasma* bacteria was 0.005–0.5%, 0.01–3%, 0.01–3% in cows, sheep, and goats, respectively (25). Spitalska et al. in 2005 investigated 100 samples of sheep blood, and 89 ticks using PCR for 16S and 18S rRNA in Shiraz City; they observed that 25% of ticks and 29% of blood samples were positive for *Anaplasma sp.* and *Ehrlichia sp.* Also, they found that 59.5% of ticks and 76% of blood samples were infected with *Babesia sp*. and *Theileria sp*. (26). A study by N’aman et al. in Isfahan City (1386) revealed some *Anaplasma*-like bodies in 75 blood smears out of 150 cow blood samples using microscopic observation. Also, they observed *An. marginale* in 50 blood samples using Nested-PCR (27). Another research in Qa’emshahr City in the province of Mazandaran performed on 98 specimens of *Ix. Ricinus* using PCR on the 16S rRNA gene revealed that 5.1% of the ticks were infected with *An. phgocytophilum*. These results can be a cause for concern for the appearance of human *An*. *granulocyte* (AGE) disease in that area (28).

In another study in Qa’emshahr using Nested-PCR on 54 *Rh. sanguineus*, 1 *Rh. bursa*, 39 *Ixodes ricinus*, 4 *Bo. annulatus*, 2 *Haemaphysalis numidiana*, and 1 *Haemaphysalis punctate*revealed that 49.5% of the ticks were positives. At the same time, 65 samples of sheep blood, 90 samples of cow blood, and 4 samples of goat blood were studied showing 43.1%, 22.2%, 25% of sheep, cows, and goats were infected respectively. The researchers reported 25% of human samples (40 blood samples) of at risk individuals from different age groups and genders were infected (24).

## Conclusion

Results of this study show preliminary data on relatively high infection rates in hard ticks collected from sheep in Kerman Province. Therefore, it is essential that health and veterinary authorities and farmers to adopt appropriate strategies to control ticks to reduce the infestation and infection rates in livestock and humans. It is also necessary that those high risk individuals including herders observe adequate health and safety measures and protect themselves against diseases transmitted by ticks. More studies are recommendded in Kerman Province as well as in other parts of Iran regarding the infection of ticks with anaplasmosis.
